# Effects of Anger and Moral Identity on the Relationship between Supervisors’ Incivility and Deviant Behavior: A Study of Public Service Officers in Republic of Korea

**DOI:** 10.3390/ijerph182010585

**Published:** 2021-10-09

**Authors:** Yong Hyun Kim, Seung Yeon Son, Seung-Wan Kang

**Affiliations:** 1 Republic of Korea Army, Daejeon 34059, Korea; rnlgks2@army.mil; 2 Graduate School of Defense Management, Korea National Defense University, Nonsan 33021, Korea; 3 College of Business, Gachon University, Seongnam 13120, Korea

**Keywords:** incivility, anger, moral identity, deviant behavior, social exchange

## Abstract

This study investigated the effects of supervisors’ incivility regarding employees’ deviant behavior, the mediating effect of anger, and the moderating role of moral identity in the relationship between incivility and deviant behavior. To test our hypotheses, we collected data from supervisor–employee dyads in South Korean military units, applying a time-lagged design, hierarchical regression, and SPSS macro. The results elicited three relevant findings. First, supervisors’ incivility was found to positively influence employees’ deviant behavior. Second, employees’ anger was confirmed to have a mediating effect between supervisors’ incivility and employees’ deviant behavior. Third, the analysis demonstrated that moral identity moderates the relationship between anger and deviant behavior, and incivility through anger has an indirect effect on deviant behavior. These findings imply that supervisors’ incivility, which is readily observed within the organization, is a harmful behavior that increases anger and deviant behavior. These findings suggest that negative leadership should be minimized and employees with high moral identity should be selected to reduce deviant behavior that harms the organization.

## 1. Introduction

Respecting and acknowledging others presupposes congenial interaction. This is applied to both peer relationships and in the supervisory management of employees. Researchers have mainly focused on effective and positive supervisory behavior; however, in actual organizations, many employees seem to be treated negatively and disrespectfully by their supervisors. In other words, incivility by supervisors frequently occurs in organizations. Supervisors’ incivility refers to a negative or deviant behavior of weak intensity that harms his/her subordinates [1]. Examples include yelling, answering phone calls insincerely, dismissing, or ignoring opinions. Jokes and sarcasm with ambiguous intentions that cause discomfort and anger for employees also constitute supervisory incivility.

Reflecting the reality of these everyday organizational circumstances, research on supervisors’ incivility in the fields of business administration and psychology began around the year 2000 [2]. Prior studies have shown that supervisors’ incivility negatively affects not only employees’ perceptions and attitudes but also their performance and creativity, which are essential to organizational success [3]. Such studies significantly contribute, providing evidence that supervisors’ incivility has a negative effect on employee attitudes, behavior, and performance, to the detriment of organizational effectiveness.

However, despite the contributions of prior studies, there are several limitations. First, while employees’ perceptions and attitudes remain important, behavior and performance are more directly related to organizational success and survival. Among them, deviant behavior is a major component of job performance and refers to discretionary behavior that causes serious problems and hinders the effectiveness of the organization [4]. As a result, interest in determinants that reduce or increase this behavior remains. Given that supervisors’ incivility is a negative behavior that violates the norms of mutual respect, there is the possibility of its triggering an employee’s retaliatory mechanism, leading to the employee’s deviant behavior. However, the research proposed and demonstrated remains very limited. Second, determining how supervisors’ incivility influences employees’ behavior and performance can offer an applicable and practical lesson, as well as a better understanding of the relationship between the constructs. Similarly, it is of theoretical and practical significance to explore the influence channels through which supervisors’ incivility links to deviant behavior. However, our current level of knowledge of the mechanisms inherent between supervisors’ incivility and deviant behavior is insufficient. Third, individual supervisor and employee differences play an important role in understanding the environment and predicting responses. Similarly, the extent to which the influence that supervisors’ behavior has on deviant behavior depends on these individual differences. In other words, individual differences can present boundary conditions that allow a more in-depth understanding of the dynamics of the effects of supervisors’ incivility. However, no research seems to have examined this consideration.

This study has three objectives. The first is to investigate the relationship between supervisors’ incivility and employees’ deviant behavior. According to social exchange theory [5], due to the mutual relationship between the supervisor and the employee, a supervisor’s incivility can trigger a negative norm of reciprocity. In particular, as the supervisor is an agent of the organization [6], employees sometimes place the blame for supervisors’ disrespectful behavior on the organization. As a result, we believe that deviant behavior that hinders supervisors’ managerial performance and damages an organization can increase. Second, we seek to reveal some of the mechanisms inherent in the relationship between supervisors’ incivility and employees’ deviant behavior. According to affective event theory [7], supervisors’ incivility is a negative event that harms employees’ wellbeing. Anger is one of the representative sentiments caused by disrespectful treatment or neglect, such as supervisors’ incivility, and can impact employees’ behavior. In this study, we endeavor to identify the mediating role of employees’ anger. 

**Figure 1:**
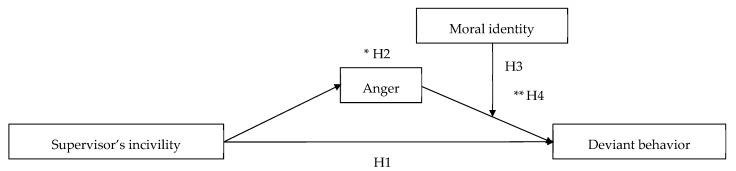
Hypothesized research model. * mediating effect, ** moderated mediating effect.

Third, we aim to verify individual differences that can act as moderators on the influence of supervisors’ incivility. Not all employees experiencing anger will engage in the same level of deviant behavior. This is because perceptions of the unethical nature of deviant behavior can vary depending on individual moral standards and values—namely, the degree of moral identity [8]. In other words, even if anger is generated by a supervisor’s incivility, employees with high moral identity are less likely to be involved in deviant behavior, a response that is inconsistent with the central concept of the moral self. This potential moderator will be explored in this study. This study’s conceptual model is illustrated in Figure 1.

This paper aims to provide practical insights into why supervisors should be concerned with their incivility in the field, in addition to increasing overall knowledge regarding the influence process by revealing how incivility in violation of reciprocity norms relates to the advent of employees’ deviant behavior. This research also seeks to enhance the understanding of the dynamics associated with the effects of supervisor incivility by identifying the boundary condition of moral identity, with clear implications for organizational efforts to promote employees’ moral identities.

## 2. Literature Review

### 2.1. Supervisors’ Incivility and Employees’ Deviant Behavior

Disrespectful words and actions can be easily identified within organizations—namely, rude people. Incivility that violates the norms of mutual respect through rude behavior without caring for others has two conceptual differences among various negative organizational behaviors [9]. First, incivility is low in intensity compared to other negative behaviors, such as abuse and aggression; if the intensity of bullying is moderate, the intensity of incivility is low [10]. Second, attacks and abuse are strongly intended to harm certain targets, whereas the intention of incivility can be ambiguous. Therefore, incivility is negative behavior with a weak intensity and ambiguous intentions that is often experienced in organizational daily life, such as teasing, ignoring, and unkindness. However, the negative effects of such behavior cannot be ignored, as the damage caused by supervisors’ incivility to employees can be detrimental to an organization. 

A supervisor may behave rudely when dealing with an employee, such as ignoring them, treating them disrespectfully, or shouting; however, the perpetrator of the incivility, the supervisor, has a position of authority that is higher than the employee [11]. Employees also continuously interact with their supervisors; therefore, even with weak intensity and ambiguous intentions, many negative effects of supervisor incivility on employee emotions, attitudes, and behavior are possible. Prior studies have demonstrated that supervisors’ rude behavior reduces job satisfaction, organizational commitment, and satisfaction with the supervisor while increasing turnover intention and work–family conflict [3]. Rude emails from non-face-to-face supervisors were also confirmed to raise employees’ negative emotions and lower positive emotions, enthusiasm for work, and performance [12]. The implication of existing studies is that supervisors’ incivility has a negative influence on employees’ perceptions, attitudes, and behavior that cannot be ignored. We assert that supervisors’ incivility is also correlated to employees’ deviant behavior.

Deviant behavior refers to behavior intended to undermine organizational norms and harm the organization or others in the organization [13]. Specifically, it is unethical behavior such as avoiding responsibilities during working hours, deliberately obstructing work, being late, or blaming colleagues or supervisors. According to existing research, organizational injustice, perceptions of unfair experiences, job dissatisfaction and insecurity, and job stress affect deviant behavior [14,15]. In particular, employees who recognize that they have been treated without dignity and respect by their supervisors often retaliate against this treatment by reducing behavior that is beneficial for the organization and engaging in harmful behavior [16]. Therefore, a significant relationship between supervisors’ incivility and employees’ deviant behavior can be inferred. According to social exchange theory, supervisors and employees form a reciprocal exchange relationship. In other words, when a supervisor is friendly or unfriendly, employees exhibit friendly or unfriendly reactions and behaviors according to reciprocity norms. However, supervisors’ incivility creates a situation of interpersonal conflict that violates mutual reciprocity, causing negative emotions and intention to retaliate [17]. In such cases, employees may increase deviant behavior that is detrimental to the organization. In addition, the supervisor is an authoritative agent of an organization; therefore, their incivility can be extended to the perception that the organization overall treats its employees disrespectfully. In such cases, employees can harm the organization represented by the supervisor with deviant behavior. 

Prior studies also find direct and indirect evidence of the relationship between supervisors’ incivility and deviant behavior. For example, when abusive supervision increases, employees’ deviant behavior also increases, and when a supervisor damages the social reputation of an employee, deviant behavior also increases. In addition, supervisors’ incivility has been negatively correlated to employees’ job performance and creativity and positively correlated to job withdrawal [18]. These studies demonstrate that disrespectful supervisory behavior is related to employees’ deviant behavior. Therefore, we propose the following hypothesis:

**Hypothesis** **1.** *Supervisors’ incivility is positively related to deviant behavior.*

### 2.2. The Mediating Role of Anger

The study of the influence of leadership behavior increases the understanding of the dynamics between variables. This research presumes that anger can explain the relationship between incivility and deviant behavior. Contemporary employees spend much of their day interacting with supervisors in the workplace, experiencing a variety of emotions. Among them, anger refers to an emotion of varying intensity, ranging from minor irritation to rage [19]. What causes employees’ anger? According to affective event theory, when employees experience emotion-stimulating events in an organization, emotions are generated accordingly, and anger is a representative emotion felt when experiencing unpleasant events [20]. In other words, disrespectful behavior by a supervisor who interacts with focal employees in everyday work life [21] is considered to violate the norms of mutual respect, causing anger. In addition, supervisors are usually recognized as authoritative organizational agents. Therefore, supervisors’ rude behavior can extend employees’ anger toward the organization that allows it to occur unchecked [22]. Prior studies also show a variety of evidence of the relationship between supervisors’ incivility and employees’ anger. For example, conflicts with supervisors were found to have a positive impact on negative emotions such as anger [23]. In addition, the supervisors’ social disturbance had a positive effect on negative emotions, and the experience of injustice in an organization raises anger [24].

According to affective event theory, negative emotions induced by negative experiences influence behavior. Therefore, anger caused by supervisors’ incivility can lead to retaliatory or negative behavior. Deviant behavior is an act of individual will that includes the expression of negative emotions and retaliatory action, posing a threat to others and organizations [15]. Therefore, a positive relationship between employees’ anger and deviant behavior is possible. Prior studies also predict and confirm the relationship between anger and deviant behavior. For example, a number of studies have confirmed negative emotions as a primary cause of deviant behavior [25]. Anger is one of the core negative emotions. In addition, anger reduced job satisfaction and task performance [26] and had a positive relationship with turnover intention. Taken together, supervisors’ incivility raises employees’ anger, which causes deviant behavior. Therefore, we propose the following hypothesis:

**Hypothesis** **2.** *Employees’ anger mediates the relationship between supervisors’ incivility and deviant behavior.*

### 2.3. The Moderating Role of Moral Identity

Prior studies demonstrate that employees’ deviance is one method of retaliating against mistreatment from an organization or supervisor [27]; however, not all employees are involved in the same level of deviant behavior. This is because the degree of deviant behavior can vary depending on an individual’s level of moral identity, a personal ethical standard for gauging unethical behavior. Identity is a self-concept or self-definition that is at the center of one’s existence and involves the perceived integrity of one’s actions [28]. Among them, moral identity is a self-concept wherein various moral beliefs are organized. If an individual’s moral standards, characteristics, and values are close to the center of self-definition, the person’s moral identity is high. According to social cognitive theory [29], moral identity is the knowledge structure that a person possesses regarding their moral characteristics and forms the basis of cognitive schemas, moral values, goals, and actions [30]. The important point is that moral identity can be based on moral and ethical judgments and has a significant impact on an individual’s perceptions of ethical or unethical behavior [31]. Therefore, a person with a high moral identity engages in actions consistent with their moral beliefs and refrains from behaviors that are inconsistent. In this study, we assert that the characteristics of moral identity can affect the relationship between anger and deviant behavior.

Employees with a high moral identity tend to be strongly aware of their responsibilities, duties, and norms and behave according to this core identity. Rather than immediately responding to environmental stimuli and their emotional state, this person carefully considers whether their actions are morally correct. Therefore, even if anger against the supervisor or organization arises, moral identity is likely to intervene and supersede the process of linking it to deviant behavior. In other words, employees with high moral identity have a relatively strong perception that deviant behavior is contrary to the norms that maintain the organization. In this case, the relationship between anger and deviant behavior is believed to weaken. 

In contrast, employees with low moral identity have relatively weak standards and values regarding whether deviant behavior, a means of retaliation due to anger, is morally wrong. They have a strong motivation to find an appropriate way to respond and retaliate against the anger caused by their supervisor’s incivility but lack a control mechanism to consider it from a moral point of view. Therefore, for employees with low moral identity, the positive relationship between anger and deviant behavior is considered relatively strong. Prior studies have provided evidence of the moderating and main effects of moral identity. For example, employees’ deviant behavior resulting from abusive supervision has been demonstrated to be stronger for employees with low moral identity [32]. It has also been confirmed that the relationship between injustice, counterproductive work behavior [33], and retaliatory behavior [34] depends on employees’ moral identity. Therefore, we propose the following hypothesis:

**Hypothesis** **3.** *Employees’ moral identity moderates the relationship between anger and deviant behavior such that a positive relationship is weaker when moral identity is high.*

Synthesizing Hypotheses 2 and 3, supervisors’ incivility raises employees’ anger and leads to deviant behavior, which can vary depending on employees’ degree of moral identity. In other words, the indirect effect of supervisors’ incivility on deviant behavior through anger will be weakened if moral identity is high. Conversely, if an employee’s moral identity is low, the indirect effect of anger related to incivility on deviant behavior can be stronger. Thus, we hypothesize the following:

**Hypothesis** **4.** *Employees’ moral identity moderates the indirect effect of supervisors’ incivility on deviant behavior via anger such that the indirect effect of supervisors’ incivility on deviant behavior via anger is weaker when moral identity is high.*

## 3. Method

### 3.1. Sample

We collected data on commissioned and non-commissioned officers from South Korean military units, which have supervisor–employee dyads in a hierarchical structure. The researcher visited the units and explained the objectives and procedures of the data collection. The researcher then distributed a survey to employees to evaluate supervisors’ incivility and their anger and their moral identities. To reduce common method bias, the deviant behavior of employees was measured with supervisors 1 month following the employees’ survey [35]. Survey questions developed in English were translated into Korean by two experts fluent in both languages and reverse-translated back into English to ensure semantic equivalence. Surveys were distributed to 250 dyads (250 supervisors and 250 employees). A total of 202 supervisor–employee dyads undertook the survey. 

Among the data that we collected from 202 dyads, we identified some unreliable responses—for instance, responses to only some of the items or selections of more than one response on a given Likert scale. After we excluded unreliable responses, we had available data for 189 dyads, for a response rate of 75.6%. The demographic statistics for supervisors revealed that the average age was 29.11 (SD = 5.08), with 183 (96.8%) males (Table 1). Regarding rank, there were 73 captains (38.6%), 49 first lieutenants (25.9%), 30 s lieutenants (15.9%), 26 master sergeants (13.8%), 10 first-class sergeants (5.3%), and 1 major (0.5%). In terms of educational levels, most supervisors (168; 88.9%) possessed 4-year bachelor’s degrees, and the rest were high school graduates (13; 6.9%) or had graduate degrees (8; 4.2%). Supervisors’ mean organizational tenure was 5.78 years (SD = 5.41). Demographic statistics for employees showed that the average age was 26.60 (SD = 5.32), with 186 (98.4%) males. Regarding rank, there were 97 staff sergeants (51.3%), 54 first-class sergeants (28.6%), 21 master sergeants (11.1%), 10 first lieutenants (5.3%), and 7 s lieutenants (3.7%). In terms of educational levels, 130 employees (68.8%) were high school graduates and the others had 4-year bachelor’s degrees (59; 31.2%). Employees’ average organizational tenure was 5.09 years (SD = 4.90). The supervisor–employee dyads had worked together for an average of 0.76 years (SD = 0.48).

### 3.2. Measures

The study used survey tools for which reliability and validity were confirmed by prior research. All items were rated on five-point Likert scales (1 = *completely* *disagree*, 5 = *completely* *agree*), excluding the control variables. Four items were adopted from Sliter et al. [36] to capture supervisors’ incivility. A sample item is “My supervisor often ignores or excludes me while at work.” Cronbach’s α of our scale was 0.94. Anger was measured with six items (α = 0.90) adopted from Watson and Clark [37]. A sample item is “angry”, regarding which employees were asked to reflect on the extent to which they felt this way when thinking about or interacting with their supervisor, as reflected in the previous study [38]. Regarding moral identity, the employees were first asked to think about the characteristics of “caring, compassionate, fair, friendly, hardworking, generous, honest, helpful, and kind.” It was then measured with five items adopted from Aquino and Reed [39] (α = 0.88). A sample item is “It would make me feel good to be a person who has these characteristics.” Deviant behavior was measured with 18 items adopted from Bennett and Robinson [4]. A sample item is “This employee spent too much time fantasizing or daydreaming instead of working.” This study aimed to focus on overall deviant behavior rather than specific targets or dimensions of deviant behavior [40]. Although Bennett and Robinson’s scale [4] has been used to measure interpersonal and organizational deviant behavior, the results of exploratory factor analysis suggest that deviant behavior is a single factor. Furthermore, the correlation between the two is very high (r = 0.85, *p* < 0.001). As with previous studies [41,42], the study was conducted in terms of overall deviant behavior. Cronbach’s α of our scale was 0.98. We controlled four employee demographic characteristics, namely age, rank, educational level, and supervisor–employee tenure.

## 4. Results

### 4.1. Measurement Validation, Correlations, and Reliability Analyses

Prior to testing the research model, we conducted a confirmatory factor analysis to test the validity of the hypothesized four-factor model. Considering the parameter-to-sample ratio [43], three indicators were created for each factor through item parceling [44]. The results of the confirmatory factor analyses are presented in Table 2. As shown, the four-factor structures were proven to be fitted with the data (χ^2^(48) = 79.20, *p* < 0.01, CFI = 0.98, GFI = 0.94, RMSEA = 0.06). In addition, we compared the hypothesized model with three alternative models. The test results confirmed that all the alternative models significantly differed from the hypothesized model and the model fit of the hypothesized model was better than other alternative models in all aspects.

The descriptive statistics, reliability, and correlations are shown in Table 3. The research variables of incivility, anger, and deviant behavior were significantly correlated and showed results consistent with the suggested study model. The results are aligned with our hypotheses. All reliability alpha coefficients of the constructs exceeded 0.7 [45].

### 4.2. Hypothesis Tests

We used hierarchical regression analysis to test the hypotheses, the results of which are shown in Table 4. Hypothesis 1 was that supervisors’ incivility has a positive effect on employees’ deviant behavior. In Model 1, incivility was found to be positively related to deviant behavior (β = 0.18, *p* < 0.05), supporting Hypothesis 1.

Hypothesis 2 forecast the mediating effect of anger on the relationship between supervisors’ incivility and deviant behavior. The mediating effect was verified using the SPSS macro presented by Preacher et al. [46]. The results are shown in Table 5. Regarding the indirect effect of supervisors’ incivility on deviant behavior via anger, 95% confidence intervals of 10,000-times repeated bootstrapping indirect effect tests did not contain zero (lower limit (LL) = 0.08, upper limit (UL) = 0.27). This finding supports Hypothesis 2.

**Figure 2:**
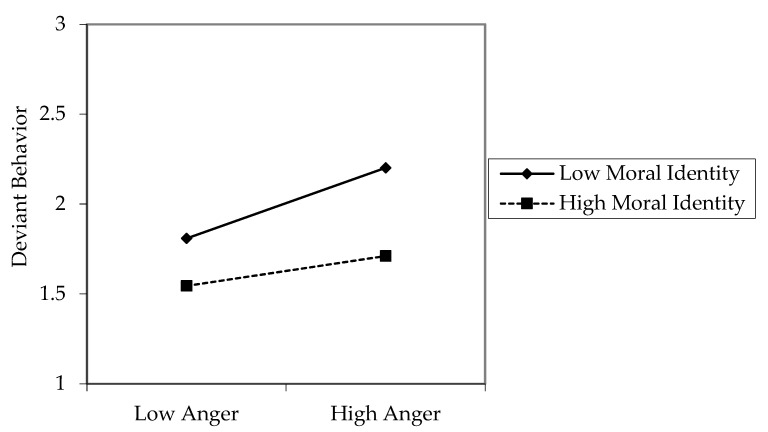
The moderating effect of moral identity on the relationship between anger and deviant behavior.

In Hypothesis 3, we proposed that moral identity moderates the relationship between anger and deviant behavior. As shown in Table 4, the interaction term of anger and moral identity significantly predicted deviant behavior (β = −0.16, *p* < 0.01; Model 4). In Figure 2, a graph distinguishes high and low moral identity groups in terms of the average value of moral identity [47]. As shown in Figure 2, when moral identity is low, the positive relationship between anger and deviant behavior is stronger. Thus, Hypothesis 3 was supported.

To test Hypothesis 4, we used the SPSS macro with 95% confidence intervals of 10,000-times repeated bootstrapping. The results are presented in Table 6. The results indicate that the indirect effect was stronger for low moral identity (conditional indirect effect = 0.26, SE = 0.07, 95% confidence interval [0.14, 0.41]) than high moral identity (conditional indirect effect = 0.11, SE = 0.05, 95% confidence interval [0.01, 0.20]), thus supporting Hypothesis 4.

## 5. Discussion

### 5.1. Study Summary

We conducted this study with three main objectives, which we addressed as follows. First, we examined the relationship between supervisors’ incivility and employees’ deviant behavior and found positive effects of incivility on deviance: when a supervisor showed incivility, the employee performed more behaviors that were harmful to the organization. Second, we explored the mediating role of anger in the relationship between incivility and deviant behavior and found that anger explained the influence of supervisors’ incivility on deviant behavior. In other words, supervisors’ incivility increased employees’ anger, which in turn resulted in their deviant behaviors. Third, our analyses confirmed that moral identity moderated the relationship between anger and deviant behavior and that incivility had an indirect effect on deviant behavior moderated through anger. Specifically, the indirect effect of supervisors’ incivility on employee’s deviant behavior through anger was weaker in employees with high moral identity.

### 5.2. Theoretical Implications

Three theoretical implications arose from this study. First, deviant behavior is a major component of job performance that is detrimental to organizations [48]. Thus, recognizing the antecedents of this behavior has many theoretical implications [49]. The hierarchical characteristics of organizations support supervisor incivility toward employees, and according to social exchange theory, supervisor incivility can cause deviant employee behavior. However, few prior researchers had examined the relationship between supervisors’ incivility and employees’ deviant behavior. With this study, we demonstrated that supervisor incivility is a harmful behavior that increases employees’ deviant behavior, which in turn has psychological and social costs for organizations. Thus, this study makes the theoretical contribution of identifying a relationship between supervisors’ incivility and deviant behavior.

Second, for this study, we assumed anger as a mediator in the relationship between supervisors’ incivility and employees’ deviant behavior, and we verified this relationship. Identifying relationships between variables that are intuitively considered relevant through systematic research contributes to the accumulation of knowledge [50]. Supervisor incivility is a negative experience for employees, and according to affective event theory, negative experiences cause the negative emotion of anger, which in turn can lead to negative behavior. Therefore, the relationship between supervisor incivility and deviant employee behavior is theoretically reasonable following emotion-related perspectives. However, we found very few research examinations of the mediating process of influence from affective perspectives. With this study, we have enhanced the understanding of the relationships between the research variables by revealing that supervisors’ incivility causes the negative emotion of anger in employees, which in turn increases their deviant behavior. In addition, we introduced affective event theory as well as social exchange theory and revealed in depth the mechanism of influence between supervisors’ incivility and employees’ deviant behavior. This offers the theoretical implication that it can be useful to combine theories to more systematically explain dynamic relationships between variables.

Third, a moral identity is the foundation of an individual’s moral values, objectives, actions, and ethical and unethical responses, and behaviors towards negative situations and emotions likely vary depending on one’s moral identity [8]. However, previous researchers have not theoretically and empirically examined the moderating function of moral identity for any effect on supervisors’ incivility. This paper contributes to a deeper understanding of the dynamics of the relationships among variables through our investigation of the effect of supervisors’ incivility on deviant behavior through anger and employees’ moral identity.

### 5.3. Practical Implications

This study provides several practical implications. First, for an organization’s survival and prosperity, it is as important to reduce negative employee behavior that damages the organization in order to encourage desirable employee behaviors. In this study, we revealed that supervisor incivility directly causes deviant behavior in employees, and we offer reasonable explanations for this relationship. We believe that managers and enterprisers who wish to increase their organizations’ effectiveness should discourage or prevent supervisor incivility toward employees. 

Second, if supervisors’ incivility stimulates employees’ deviant behavior, it is important to understand why, and it is also meaningful to understand the conditions that decrease the negative influence of supervisors’ incivility. In this study, supervisor incivility increased deviant employee behavior through employee anger, but the relationship decreased with greater employee moral identity. This study provides implications for human resource management regarding employees who should be hired and the types of education and training that can minimize employees’ anger and deviant behavior in response to supervisor incivility [51]. 

### 5.4. Limitations and Calls for Further Studies

This study has several limitations. First, we inferred causal relationships among variables based on prior research and theoretical backgrounds, attempting to minimize logical leaps by using a time-lagged design. However, we could not establish causal relationships solely with this study; for example, if an employee shows high deviant behavior, his or her supervisor is likely to express increased incivility. Therefore, it is necessary to verify causal relationships through longitudinal or experimental studies in the future. Second, based on affective event theory, we demonstrated anger as an influence in the relationship between incivility and deviant behavior, but there could be a variety of other mechanisms at play. For example, a supervisor’s disrespect could undermine an employee’s job satisfaction and increase deviant behavior, or a supervisor’s incivility could diminish employees’ perceptions of supervisory or organizational support. In addition, previous studies demonstrated that organizational commitment and job attachment, which are threatened by supervisors’ incivility, were related to deviant behavior. Future researchers should verify these more diverse mechanisms of the influence of incivility. 

Third, this study involved members of a military organization of the Republic of Korea. Not only is there necessarily frequent interaction between supervisors and employees in military settings, but deviant behavior is high in military organizations. Nevertheless, the uniqueness of Korea’s military could limit the generalizability of this study’s results to some extent. In addition, the sample size of this study was relatively limited in that it might be unusual for a sample to comprise more than 90% men. In the future, it would be desirable for researchers to study diverse organizations in different countries to confirm or refute the external validity of this study. Finally, in this study, we selected individual moral identity as the moderating variable based on prior research and theories, but many variables can affect supervisor incivility. For example, personality traits such as agreeableness, extraversion, and core self-evaluation can affect the incivility–anger–deviant behavior relationship. Future researchers should study more potential boundary conditions. 

## Figures and Tables

**Table 1:** Demographic statistics.

	Supervisors		Employees	
	Frequency	Percent (%)	Frequency	Percent (%)
Age (year)				
21–30	133	70.4	156	82.5
31–40	47	24.9	31	16.4
40–50	9	4.7	2	1.1
Gender				
Male	183	96.8	186	98.4
Female	6	3.2	3	1.6
Rank				
Major	1	0.5		
Captain	73	38.6		
First lieutenant	49	25.9	10	5.3
Second lieutenant	30	15.9	7	3.7
Master sergeant	26	13.8	21	11.1
First-class sergeant	10	5.3	54	28.6
Staff sergeant			97	51.3
Education				
Graduate degree	8	4.2		
4-year bachelor’s degree	168	88.9	59	31.2
High school graduate	13	6.9	130	68.8

Notes: *n* = 189.

**Table 2:** Model fit statistics for measurement models.

Model	χ^2^ (*df*)	CFI	GFI	RMSEA	∆χ^2^ (*df*)
Hypothesized four-factor model (INC, MI, anger, DB)	79.20 (48) **	0.98	0.94	0.06	
Three-factor model (INC and anger, MI, DB)	261.74 (51) ***	0.89	0.79	0.15	182.54 (3) ***
Two-factor model (INC and anger, MI and DB)	585.11 (53) ***	0.72	0.65	0.23	505.91 (5) ***
Single-factor model	1068.44 (54) ***	0.46	0.50	0.32	989.24 (6) ***

Notes: INC, incivility; MI, moral identity; DB, deviant behavior; CFI, comparative fit index; GFI, goodness of fit index; RMSEA, root mean square error of approximation; the Chi-square difference for each model reflects its deviation from the four-factor model. ** *p* < 0.01, *** *p* < 0.001.

**Table 3:** Means, standard deviations, correlations, and reliabilities.

Variable	Mean	SD	1	2	3	4	5	6	7	8
1. Age	26.60	5.32								
2. Rank	2.01	1.60	0.29 ***							
3. Education	1.31	0.47	0.38 ***	0.58 ***						
4. Tenure with supervisor	0.76	0.48	0.01	−0.18 *	−0.10					
5. Incivility	1.54	0.69	−0.21 **	−0.09	−0.12	0.11	(0.94)			
6. Anger	1.22	0.45	−0.13	0.01	0.01	0.15 *	0.69 ***	(0.90)		
7. Moral identity	4.58	0.54	0.15 *	0.10	0.13	−0.05	−0.15 *	−0.08	(0.88)	
8. Deviant behavior	1.25	0.48	−0.20 **	−0.13	−0.09	0.03	0.21 **	0.32 ***	−0.43 ***	(0.98)

Notes: *n* = 189. Reliability alpha (α) coefficients are reported in diagonal. * *p* < 0.05, ** *p* < 0.01, *** *p* < 0.001.

**Table 4:** Results of regression analyses.

Variable	Deviant Behavior			
	Model 1	Model 2	Model 3	Model 4
Age	−0.15	−0.13	−0.09	−0.09
Rank	−0.09	−0.11	−0.11	−0.11
Education	0.04	0.01	0.03	0.02
Tenure with supervisor	0.00	−0.03	−0.04	−0.06
Incivility	0.18 *	−0.07	−0.12	−0.14
Anger		0.36 ***	0.37 ***	0.37 ***
Moral identity			−0.40 ***	−0.39 ***
Anger × moral identity				−0.16 **
∆*R*^2^	0.07	0.06	0.15	0.03
∆*F*	2.94 *	13.52 ***	38.28 ***	7.88 **

Notes: *n* = 189. Standardized regression coefficient betas are presented. * *p* < 0.05, ** *p* < 0.01, *** *p* < 0.001.

**Table 5:** Results of indirect and conditional indirect effect analyses.

Mediator	Indirect Effect	SE	95% LLCI	95% ULCI
Anger	0.17	0.05	0.08	0.27

Notes: *n* = 189. Bootstrap sample size = 10,000. LLCI, lower limit confidence interval; ULCI, upper limit confidence interval.

**Table 6:** Results of indirect and conditional indirect effect analyses.

Moderator	Indirect Effect	SE	95% LLCI	95% ULCI
Moral identity −1 SD	0.26	0.07	0.14	0.41
Moral identity +1 SD	0.11	0.05	0.01	0.20

Notes: *n* = 189. Bootstrap sample size = 10,000. LLCI, lower limit confidence interval; ULCI, upper limit confidence interval.

## Data Availability

Not applicable.
